# Supplementary material for: Comparative Phytochemical, Antioxidant, and Hemostatic Studies of Extract and Four Fractions from Paulownia Clone in Vitro 112 Leaves in Human Plasma
Source: Molecules. 2020 Sep 23;25(19):4371. doi: 10.3390/molecules25194371 (PMC7583007; doi:10.3390/molecules25194371)
Supplement: Supplementary file 1 [file molecules-25-04371-s001.pdf]

molecules

MDPI

Supplementary materials

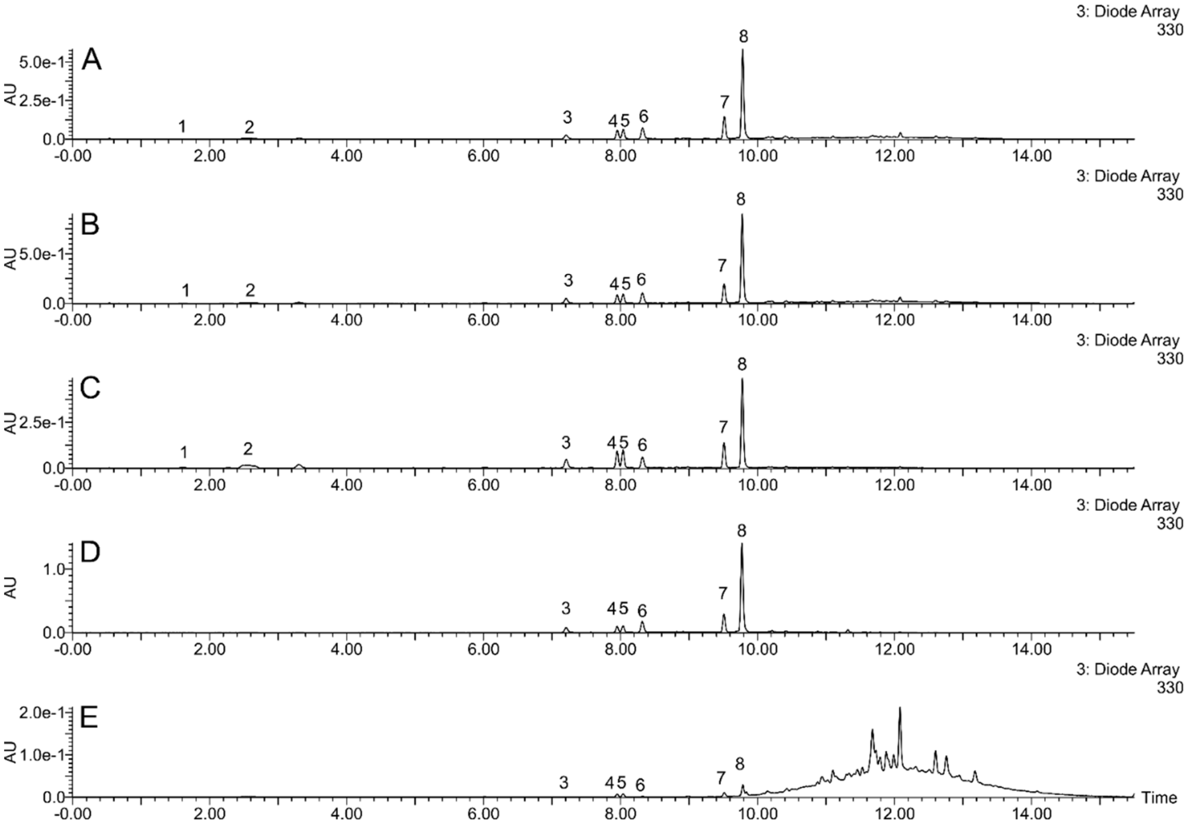

Figure S1. UHPLC-UV chromatograms (λ = 330 nm) of the Paulownia Clone in Vitro 112 leaf extract (**A**), fraction A (**B**), fraction B (**C**), fraction C (**D**), and fraction D (**E**). 1—aucubin/7-hydroxytometoside; 2—caffeic acid-Hex-dHex; 3—luteolin-HexA-HexA; 4 & 5—hydroxyverbascoside; 6—apigenin-HexA-HexA; 7—metoxyverbascoside; 8—verbascoside. HexA—hexuronic acid.

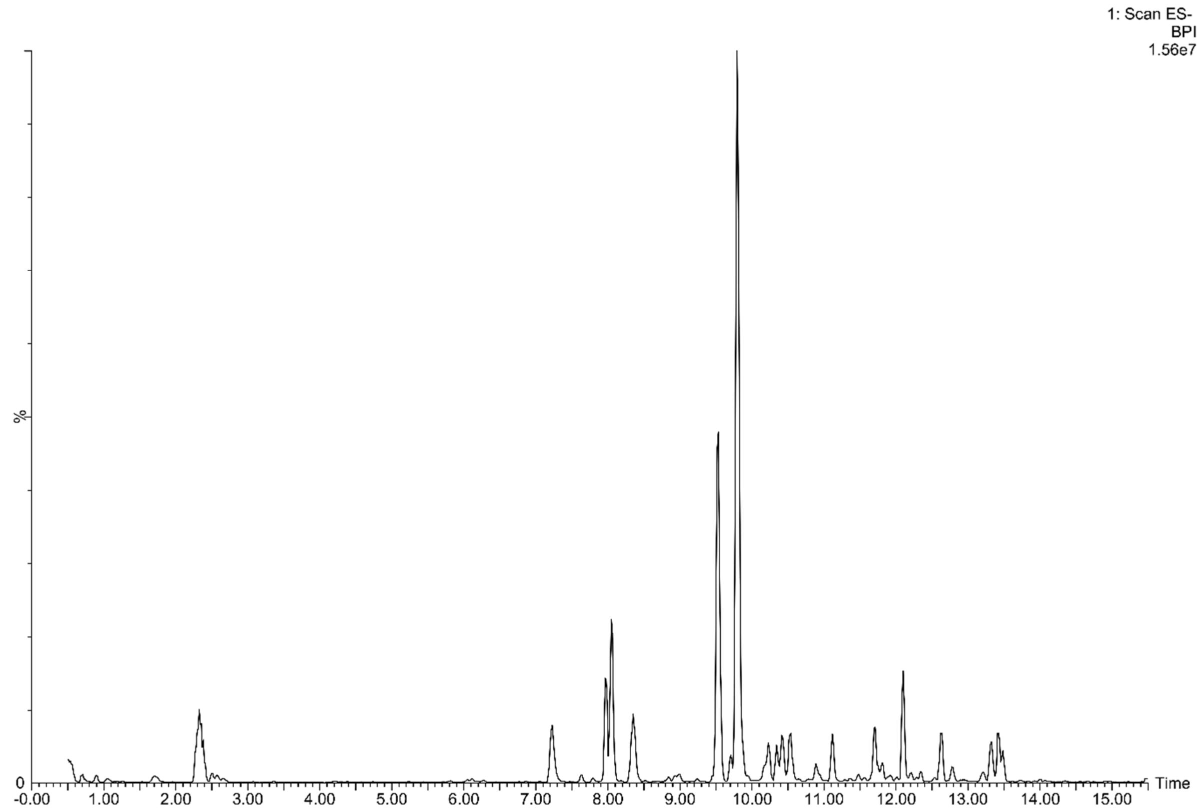

Figure S2. UHPLC negative ion base peak chromatogram of the Paulownia Clone in Vitro 112 leaf extract.
